# Development of the Musi-CI Training, A Musical Listening Training for Cochlear Implant Users: A Participatory Action Research Approach

**DOI:** 10.1177/23312165231198368

**Published:** 2023-09-12

**Authors:** Joke Veltman, Marjo J.M. Maas, Cilia Beijk, Adinda Y.M. Groenhuis, Huib Versnel, Constance Vissers, Wendy J. Huinck, Alexander E. Hoetink

**Affiliations:** 1 Behavioural Science Institute, 6029 Radboud University, Nijmegen, the Netherlands; 2 Stichting Musi-CI, Breda, the Netherlands; 3 Radboudumc IQ Healthcare, Nijmegen, the Netherlands; 4 6032 HAN University of Applied Sciences, Nijmegen, the Netherlands; 5 Department of Otorhinolaryngology and Head and Neck Surgery, Radboud university medical center, Nijmegen, the Netherlands; 6 Department of Otorhinolaryngology and Head & Neck Surgery, University Medical Center Utrecht, 8125 Utrecht University, Utrecht, the Netherlands; 7 UMC Utrecht Brain Center, 8125 Utrecht University, Utrecht, the Netherlands; 8 Royal Dutch Kentalis, Sint-Michielsgestel, Netherlands; 9 Donders Institute for Brain, Cognition and Behavior, 6029 Radboud University, Nijmegen, the Netherlands

**Keywords:** cochlear implants, rehabilitation, musical training, auditory training, thematic analysis

## Abstract

A cochlear implant (CI) is a prosthesis that allows people with severe to profound hearing loss to understand speech in quiet settings. However, listening to music presents a challenge to most CI users; they often do not enjoy music or avoid it altogether. The Musi-CI training course was developed for CI users with the goal of reducing music aversion and improving music enjoyment. A consortium was established consisting of a professional musician with CI, CI rehabilitation professionals and researchers. Participatory action research (PAR) was applied to develop and evaluate the training experiences, collaborating with 37 CI users during three cycles of eight training sessions, each held over a period of 3 months. Input and feedback were collected after each training session using questionnaires, observations and focus group interviews. Almost all participants (86%) completed the training. After completing the training a large majority of participants reported increased music appreciation, increased social participation in musical settings and a positive impact on general auditory perception. The resulting Musi-CI training programme focuses on music listening skills, self-efficacy, and self-motivation. It consists of exercises intended to strengthen attention and working memory, to improve beat and rhythm perception (with online rhythm exercises) and exercises to distinguish timbre of instruments and emotion in music. A Melody Game was developed to improve pitch and melodic contour discrimination.

## Introduction

A cochlear implant (CI) is an inner ear prosthesis that allows people with severe to profound hearing loss to detect and recognise environmental sounds and to understand speech in quiet settings. CI rehabilitation in the Netherlands focuses primarily on restoring speech recognition. Over an intensive period of adjusting processor settings and attending auditory training, CI users typically relearn this ability 3–12 months post-implantation. Most CI users achieve speech recognition in easy listening conditions, e.g. in a quiet environment or with just one or two speakers in proximity (Gfeller et al., 2019a, 2019b; Limb, 2006). In difficult listening situations, such as with considerable background noise and/or reverberation or in phone calls with limited bandwidth, speech recognition remains challenging for many CI users. Some users achieve a satisfactory level of speech recognition and experience little hindrance in such environments, while others are affected so severely that participation seems unattainable (Swaminathan et al., 2015).

Listening to music can present an immense challenge when the listener has experienced music enjoyment prior to implantation (Chari et al., 2020; Jiam et al., 2019; Magele et al., 2022; Riley et al., 2018). This group of CI users often cannot fully enjoy music, or at worst, feel sheer aversion to it (Hutter et al., 2015). Many CI users withdraw from settings where music is being played, which can lead to social isolation, feelings of loss and grief and a reduced quality of life (Magele et al., 2022; van Besouw et al., 2014). CI users often perceive music as out of tune, dissonant, tinny, indistinct, having weak bass tones and lacking emotions (Jiam et al., 2017). In general, sounds processed through a CI are perceived in a distorted manner (Dorman et al., 2020; Gfeller et al., 2015; Peters et al., 2018). This distortion may not impede speech recognition in quiet settings but does significantly affect music perception.

Why is music so important to us? Music is omnipresent and plays a significant role in our daily lives (Riley et al., 2018). The intrinsic value of music lies in its power to connect people in social situations (whether joyful, sorrowful, angry or peaceful) and at several levels of interaction, leading to increased interconnectedness and strengthened interpersonal bonds (Cross, 2009). In this way music contributes to our psychological and social sense of well-being (Dritsakis et al., 2017; Hutter et al., 2016; Magele et al., 2022; Riley et al., 2018; van Besouw et al., 2015).

Although musical listening training may have a positive influence on music perception and appreciation, little attention is paid to music perception and appreciation during regular CI rehabilitation sessions due to insufficient available time and lack of specialised expertise. According to Looi and She (2010) incidental exposure to music does not improve music perception. Only sufficient exposure to music in an active rewarding training course with relevant exercises may contribute to improved music enjoyment (Driscoll, 2012; Gfeller et al., 2015) and potentially leading CI users towards re-engaging with music (van Besouw et al., 2014). Magele et al. (2022) argue that it takes time for CI users to become habituated to the sound of music for them to enjoy it. This time investment could lead to improved music appreciation, increased social participation and thus a better quality of life (Dritsakis et al., 2017; Gfeller et al., 2019b).

Reported studies indicate a diversity in training formats, ranging from approximately 4 weeks (Chari et al., 2020; Driscoll, 2012; Fuller et al., 2018) to approximately 16 weeks of sessions (Looi & She, 2010; Magele et al., 2022; Vandali et al., 2015) and varying from in-person lessons (Hutter et al., 2016; Magele et al., 2022; Plant, 2015) to online computer-based exercises (Driscoll et al., 2009; Jiam et al., 2019; Smith et al., 2017; van Besouw et al., 2015; Vandali et al., 2015; Vickers et al., 2021). The literature recommends a variety of exercises to contribute to greater music enjoyment, such as practising with simple arrangements of familiar, monophonic music in a slow tempo, subsequently expanding in complexity (Prevoteau et al., 2018), adding visual cues (Chari et al., 2020; Plant, 2015; Vickers et al., 2021), working with the timbre of instruments (Driscoll, 2012), and practising with melodic contours (Chari et al., 2020; Fuller et al., 2018). Working with selective, alternating, and divided attention is recommended as well (Smith et al., 2017), along with focusing on problem-solving skills (Gfeller et al., 2019b). Gfeller et al. (2015) described an analytic approach (listening for increasingly difficult contrasts in pitch and timbre) and a holistic approach (listening to complete songs) as versatile methods for musical listening training. The literature on music perception with CIs generally shows broad agreement that timbre and pitch are the most challenging aspects of music perception (Driscoll et al., 2009; Hsiao & Gfeller, 2012; Jiam et al., 2018; Petersen et al., 2009; Riley et al., 2018; Vandali et al., 2015; van Besouw et al., 2015). It also illustrates the advantage of in-person training, i.e. the opportunity to bring CI users in contact with their peers to discuss acceptance of the new sound of the CI (Hutter et al., 2016) and to promote social interaction (Fuller et al., 2018). Computer-based training programmes are generally considered as supportive tools, facilitating practice outside the clinical context (Jiam et al., 2019; Smith et al., 2017; van Besouw et al., 2015).

The aim of the current study was to develop a musical listening training course involving CI users at every step of the development process to define the goals. We established a consortium consisting of CI rehabilitation professionals (CB, AG), researchers (AH, WH, MM, HV, Stichting Musi-CI, and a professional musician with CI (JV), allowing her unique knowledge to inform clinical practice. The consortium functioned as an interdisciplinary research and development team with a rich diversity in expertise in the fields of audiology, auditory neuroscience, speech and language therapy, neuropsychology, education, training design and music.

The training was developed using a PAR process; the research was conducted in 3 cycles in conformance with the framework of the National Institute for Health Research (NIHR) and Medical Research Council (MRC) (Skivington et al., 2021). In order to accommodate the needs and wishes of CI users it is important to develop training interventions in collaboration with them as co-creators (Abma et al., 2019; Looi & She, 2010; Vickers et al., 2021). An example of a participatory design approach that was successfully implemented is a computer-based musical training developed by van Besouw et al. (2016), which ensured its relevance to end-users. Our training approach aimed to reduce music aversion and enhance music enjoyment. Our end-users were tasked with providing feedback on the training design at different stages and to help determine how the programme should be run. The resultant training materials of the Musi-CI training will be provided alongside clinical resources as a training guide for CI rehabilitation professionals.

Our questions were as follows: what key elements should be included in a musical listening training for CI users that properly accommodate their needs, wishes and abilities? How do CI users experience the Musi-CI training and what impact, if any, do they perceive on their music listening abilities?

## Methods

### Participatory Action Research and NIHR-MRC Framework

We applied a participatory action research (PAR) approach to develop the Musi-CI training and evaluate participants’ experiences (Abma et al., 2019; Bergold & Thomas, 2012; van Besouw et al., 2015). This approach allows CI users and CI rehabilitation professionals to co-create the cycles of development, testing, and evaluation of the training intervention. By developing the Musi-CI training with CI users, it more accurately reflects their needs, wishes, skills, and individual musical experiences (Abma & Broerse, 2010; Looi & She, 2010). All elements of the musical listening training were evaluated by the Consortium members, to identify which elements of the training course are successful in which circumstances.

We used the framework for the development and evaluation of complex interventions from the NIHR-MRC. This framework aims to help researchers design and conduct research with a diversity of stakeholder perspectives and appropriate choice of methodology. It contains four phases: in **phase 1**, the development phase, the intervention is designed by explicating the programme theory. In **phase 2**, the modifying phase, the intervention – including its programme theory – is tested and evaluated to modify the intervention in **phase 3**, the evaluation phase, the effectiveness of the intervention is assessed e.g., by clinical trial, which may lead to **phase 4**, the implementation phase. The present study can be situated in phase 2 (Skivington et al., 2021) in which training experiences were evaluated. Training outcomes will be evaluated in subsequent controlled validation studies (phases 3 and 4).

### Developing the Musi-CI Training: NIHR-MRC Framework Phase 1

The experiential and professional knowledge of JV as Master of Music, pianist and CI user served as the foundation of the intervention. JV retrained her music-listening ability with the CI by herself, motivated by the wish to resume her professional activities. She based the training content and programme structure on a professional framework, underpinned by the existing theory on music education and Neurologic Music Therapy (NMT) (see Supplementary File 1). NMT is an evidence-based training method with some exercises specifically designed for CI users. Basic musical elements are used: beat (the basic, repeating pulse of music) and rhythm (Hsiao & Gfeller, 2012; Magele et al., 2022; Prevoteau et al., 2018), pitch and melody (Jiam et al., 2019; Magele et al., 2022; Prevoteau et al., 2018; Vandali et al., 2015), and timbre of instruments (Driscoll, 2012; Hsiao & Gfeller, 2012; van Besouw et al., 2015).

The consortium empowered JV to apply a research perspective to the training programme. By explicating JV's experiential knowledge and comparing this knowledge to the existing literature on learning and behavioural change, a conceptual framework (see Supplementary File 2) was developed to guide the conduct and the evaluation of the intervention. The role of JV was twofold: on one hand to conduct the Musi-CI training and coach the participants according to the conceptual and professional framework, on the other hand, to act as a role model for these participants.

### The Content of the Musi-CI Training, Design 1

Based on practical experiences of the CI rehabilitation professionals, the Musi-CI Consortium estimated that 8 lessons of 2 h delivered over a period of 3 months would sufficiently provide CI users with an adequate foundation to start listening to music again. We hypothesised that a) participants would need this 3-month period to acclimate to the sound of music, develop new routines in music listening, cope with feelings of music loss and overcome their music aversion (Magele et al., 2022), and that b) a 3-month training period would be practicable for participants and future trainers. The literature is not conclusive on the optimal duration of a training period; the shortest trainings in the literature had a duration of four weeks (Chari et al., 2020; Smith et al., 2017), the longest spanned six-month (Petersen et al., 2009). In the interest of pragmatism we opted for a timeframe approaching the median of the range that the literature provides.

The Musi-CI training design 1 consists of music listening exercises (attentive listening to different genres of music) and music making exercises (clapping exercises and playing with metallophone keys) (Fuller et al., 2018; Gfeller et al., 2019a, 2019b; Patel, 2014; Petersen, 2009; Plant, 2015) to compose a diverse and rewarding training course. See Table 1 for an overview of design 1.

**Table 1.** Content of the Musi-CI Training, Design 1

EXCERCISES	CONTENT of DESIGN 1	REFERENCES
Music Listening exercises	Listening to small differences in timbre of instruments, beginning with solo instruments and gradually increasing the number of instruments.	Driscoll et al., 2009; Gfeller et al., 2015; Gfeller et al., 2019a; Patel, 2011; Riley et al., 2018.
	Identifying emotional responses to different musical moods in well-known pieces of varied, mainly classical, quiet music.	Gfeller et al., 2005; Gfeller et al., 2019b; Magele et al., 2022; Plant, 2015
	Listening to small differences in rhythm and melody, based on exercises from Aural Training in Practice of the ABRSM (Associated Board of the Royal Schools of Music).	https://www.abrsm.org
Music Making exercises	Clapping exercises with symbols representing movements, adapted from the Ronnie Gardiner Method (RGM).	Pohl, 2018. www.ronniegardinermethod.com
	Singing exercises with well-known songs and rounds.	Gfeller et al., 2005; Gfeller et al., 2019b; Magele et al., 2022; Plant, 2015
Homework	A brief summary of the lessons, accompanied by website links to the music used. The clapping exercises were documented, as well to provide participants with the opportunity to do them at home for enhanced training effect.	Based on theory that states that repetition is important (Patel, 2014) and that (nearly) daily training could improve music perception (Driscoll et al., 2009; Galvin et al., 2007; Gfeller et al., 2015; Magele et al., 2022).

In accordance with phase 2 of the NIHR-MRC framework the Musi-CI training design 1 was tested, evaluated, and modified with CI users.

### Testing and Modifying the Musi-CI Training, NIHR-MRC Framework Phase 2

**Figure 1.**
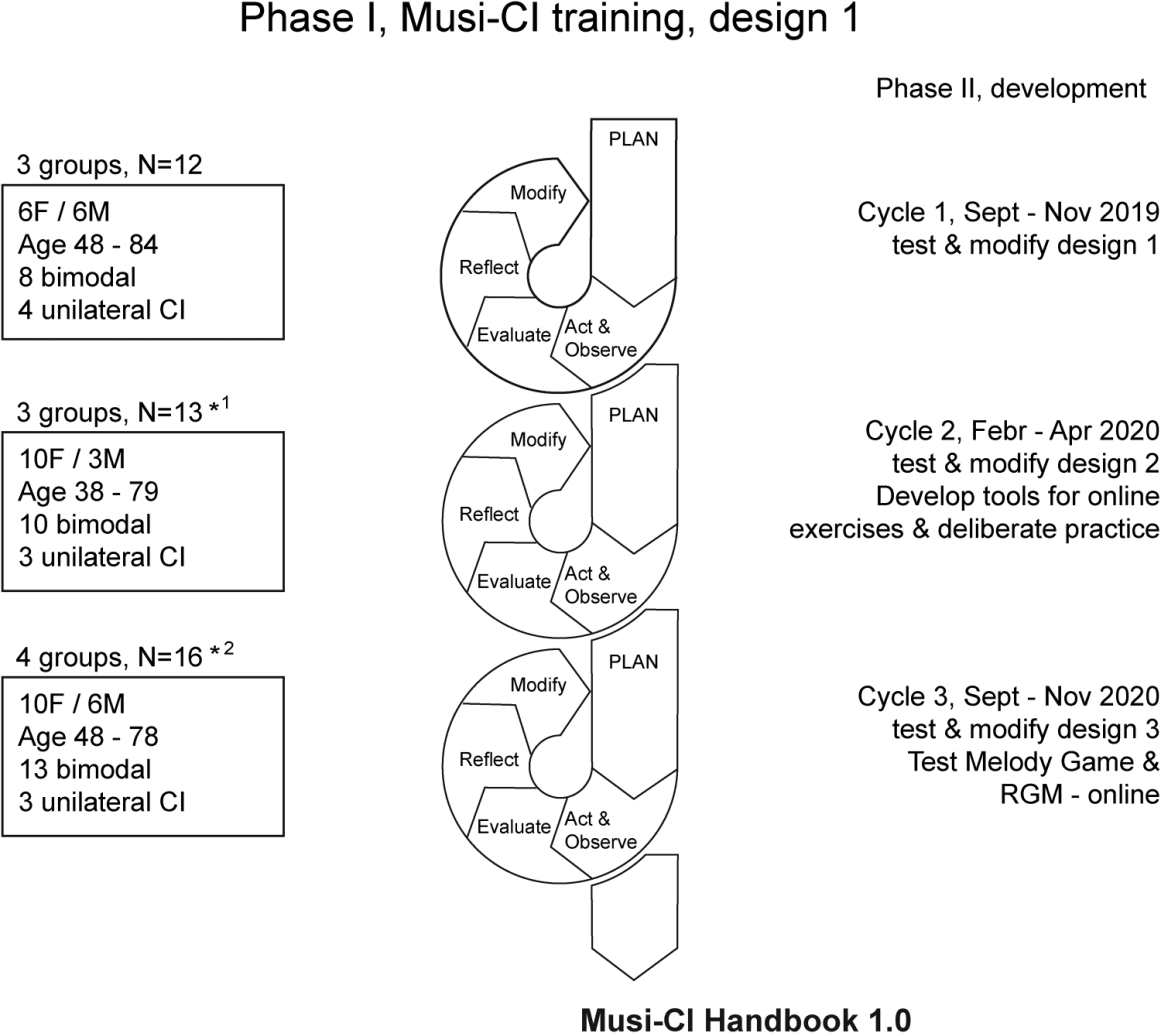
PAR cycle for modelling the Musi-CI training adopted from Van Besouw et al. (2015) and adapted to this study. Each cycle consisted of 8 lessons and was conducted within a 3-month period. ★ ^1^ one participant from cycle 1 repeated the training. ★^2^ two participants from cycle 1 and 2 participants from cycle 2 repeated the training.

We tested and improved the Musi-CI training in three PAR cycles. The first cycle started in September 2019, the second in February 2020 and the third in September 2020. The PAR cycles consisted of planning the training, conducting and observing the training, conducting same day evaluations of the training experiences, reflecting on observations and evaluation results, and modifying the training for the next cycle with close collaboration between the participants and the Musi-CI consortium. Figure 1 shows the phases of the PAR process with CI user participants and the cycles of the Musi-CI training.

### Recruitment of Participating CI Users

We recruited 37 adults with a postlingual onset of deafness. They were informed about the Musi-CI training via the OPCI website (Independent Platform for Cochlear Implantation), the Radboudumc CI user forum or were invited by a CI speech and language therapist (AG) of UMC Utrecht. All CI users were asked to register via the Musi-CI website (www.musi-ci.nl). Participants were informed about the training process and were asked to sign an informed consent, grant permission for the anonymised use of their data and video/audio registrations and comply with the Dutch rules of General Data Protection Regulation. The UMC Utrecht medical research ethics committee states that the training does not fall within the remit of the Medical Research Involving Human Subjects Act (WMO).

We aimed to acquire 30 participants, 10 per cycle, divided into small groups of 3 to 5 individuals. The participants were informed of the training's intensity at the beginning of each cycle. They were expected to attend at least 6 out of 8 two-hour lessons, to be physically able to actively participate in the exercises, and to complete a minimum of 10 min of homework per day. To guarantee adequate input and feedback, participants were asked to fill out several questionnaires regarding all aspects of the Musi-CI training as well as evaluation forms inquiring into their perception of the value, intensity and workload of the training. They were also invited to attend a closing focus group interview. A diverse group of postlingually deafened CI users, ranging in age from 38 to 84, participated in the Musi-CI project. Table 2 details the participants’ characteristics.

**Table 2.** Participants Characteristics.

Nr.	Cycle 1/2/3	Male/Female	Age (years)	Bimodal, unilateral CI	CI use (years)	Musical experience pre-implan-tation	Phone call	Completed training
1	C1	F	71	Bimodal	3	Passive	Yes, difficult	Yes
2	C1	F	65	Bimodal	4	Active	Yes, difficult	Yes
3	C1	M	62	Unilateral CI	5	Active	Yes, difficult	Yes
4	C1	M	67	Unilateral CI	3	Active	No	Yes
5	C1	F	67	Unilateral CI	6	Active	No	Yes
6	C1, 3	F	63	Unilateral CI	<1	Passive	No	Yes
7	C1	M	72	Bimodal	1	Passive	Yes, difficult	Yes
8	C1	M	69	Bimodal	<1	Active	Yes, easy	Yes
9	C1, 3	F	48	Bimodal	1	Passive	Yes, difficult	Yes
10	C1	M	62	Bimodal	<1	Active	Yes, easy	Yes
11	C1	M	84	Bimodal	2	Active	Yes, easy	No
12	C1,2,3	F	72	Bimodal	1	Active	Yes, easy	Yes
13	C2	F	38	Bimodal	<1	Active	No	No
14	C2	F	67	Bimodal	<1	Passive	Yes, easy	No
15	C2	F	69	Unilateral CI	7	Active	Yes, difficult	Yes
16	C2	F	79	Unilateral CI	1	Passive	No	No
17	C2	F	48	Bimodal	1	Active	Yes, difficult	Yes
18	C2	F	67	Bimodal	1	Active	Yes, difficult	No
19	C2	M	75	Bimodal	4	Active	Yes, easy	Yes
20	C2	F	66	Bimodal	2	Active	Yes, easy	Yes
21	C2	F	68	Unilateral CI	12	Passive	No	Yes
22	C2	F	65	Bimodal	3	Active	Yes, difficult	Yes
23	C2	M	54	Bimodal	1	Active	Yes, difficult	Yes
24	C2	M	68	Bimodal	<1	Passive	Yes, difficult	Yes
25	C3	F	78	Unilateral CI	9	Active	Yes, difficult	Yes
26	C3	F	70	Bimodal	1	Passive	Yes, difficult	Yes
27	C3	F	61	Bimodal	2	Active	Yes, difficult	Yes
28	C3	M	60	Bimodal	1	Active	Yes, difficult	Yes
29	C3	M	60	Bimodal	5	Passive	No	Yes
30	C3	M	59	Bimodal	3	Active	No	Yes
31	C3	F	48	Bimodal	1	Active	Yes, difficult	Yes
32	C3	F	68	Bimodal	1	Active	Yes, easy	Yes
33	C3	F	59	Bimodal	9	Active	Yes, easy	Yes
34	C3	M	62	Bimodal	3	Active	Yes, easy	Yes
35	C3	F	62	Bimodal	<1	Active	Yes, difficult	Yes
36	C3	M	74	Bimodal	<1	Passive	No	Yes
37	C3	M	63	Bimodal	1	Active	Yes, difficult	Yes

*Note. C1 = Cycle 1, C2 = Cycle 2, C3 = Cycle 3. Bimodal: CI and hearing aid; unilateral CI is CI-only. Musical experience pre-implantation: active indicates instrumental lessons, choir singing, playing in a group, mostly before onset of hearing loss. Passive is only listening to music. Phone call: being able to make a phone call.*

### Data Collection with Questionnaires, Interviews, and Direct Observation

We gathered information from the participating CI users at the beginning of each PAR cycle to determine their musical needs and wishes, between lessons to obtain feedback, and at the end of each PAR cycle to explore their experiences. Information garnered between cycles was used to modify the training for the next cycle. The end evaluation was used to explore the impact of the Musi-CI training on changes in listening experiences and social participation.

*Start-End questionnaire:* To help participants set their own goals for the Musi-CI training, at each PAR cycle the participants received a Start-questionnaire with 2 questions: 1. ‘Please describe three goals you wish to achieve during the Musi-CI training’ 2. ‘What is your level of motivation for the Musi-CI training?’ At the end of each PAR cycle, we returned the questionnaires that were filled in at the beginning to each participant and prompted them to describe the outcomes of their goals and their motivation.

*In-between evaluation with CI users.* In order to obtain feedback from the participating CI users, we used questionnaires containing open-ended questions on the different training components to evaluate each training session. Remarks from participants regarding their experiences with the training and its impact on their daily lives were recorded in a log book.

*In-between evaluation with CI rehabilitation professionals.* To obtain feedback from CI rehabilitation professionals, each training session was observed by CB, AG, or their direct colleagues, using pre-structured observation forms. After each session JV and the observants reflected collectively on participant feedback and on their own observations, e.g. how participants struggled with music aversion and failed to recognise previously familiar melodies.

*Final evaluations with CI users.* To identify the potentially effective elements of the Musi-CI training and their impact on music listening experiences and social participation, focus groups were conducted with CI users after each PAR cycle. These focus groups were conducted by consortium members (CB, AG, HV) using an interview guide. This guide contained the following questions: 1) How did you perceive the training?, 2) what were its strengths and weaknesses?, 3) what are your suggestions for improvement? JV was not involved in these interviews to avoid bias and guarantee impartiality of the findings. All end-evaluation interviews were video-recorded and transcribed verbatim. All questionnaires, evaluation forms and interview guides are published on the website of Stichting Musi-CI: www.musi-ci.nl.

### Data Analysis

*Start-End questionnaires.* Qualitative data on participants’ personal goals before the training were studied and analysed by JV and MM. Similar personal goals were coded and codes were merged into meaningful goal categories. We calculated quantitative data on goal attainment upon completion and described frequencies of goal attainment per goal category (see Table 3).

**Table 3.** Answers of the 37 Participants on the Start- and End-Questions in Categories. Numbers for Post-Training Attained Goals do not Necessarily Add up to Numbers Pre-Training, see Text for Explanation.

	Answer categories of the start-end questionnaires	Personal goals pre-training	Attained goals post-training
1.	**Music appreciation and recognition**	31	24, 5 ∼
	recognising familiar melodies	6	- 3 ∼
	recognising the sound of instruments	2	2
	more appreciation, more enjoyment	19	18, 2 ∼
	perceiving the beat to be able to dance again	4	4
2.	**Uptake of musical activities**	22	12, 4 ∼
	having the courage to listen again	5	2
	listening to music at home	4	4, 1∼
	listening to new music	7	2, 3∼
	singing and/or playing your instrument	6	4
3.	**Uptake of social activities**	5	3, 1 ∼
	Going to concerts/festivals/parties again	5	3, 1 ∼
4.	**Self-reported improvement of discrimination of speech and lyrics**	7	5 3 ∼
	understanding the lyrics while listening	4	3, 2 ∼
	understanding speech better	3	2, 1∼

The sign ∼ indicates that personal goals were attained partially. See text for further explanation.

*In-between evaluations.* Written feedback from CI users and observations by CI rehabilitation professionals (CB, AG and colleagues) were studied and analyzed by CB, AG, JV and MM during the training in an ongoing process to improve the training stepwise (Figure 1). They searched for similarities and patterns in participant experiences and discussed the findings. Data were collapsed into meaningful categories relevant to improve the training for the next cycle.

*Final evaluations.* To explore the participants’ experiences and their perceived impact on music enjoyment, JV and MM, who were not involved in the interviews, analysed transcripts of final evaluations using thematic analysis (Vaismoradi et al., 2016). This is a standardised, descriptive approach to the data. It provides a descriptive, in-depth report of the findings. JV and MM studied the first focus group transcript individually and coded pieces of text relevant to the research questions in Word Microsoft Office 365. They developed a code book by comparing and discussing their particular codes. All transcripts were coded accordingly. By continuously discussing and comparing codes they identified patterns and merged codes into meaningful categories. They triangulated the data from in-between evaluations and end-evaluations to developed themes, representing the potentially effective elements of the Musi-CI training and their impact on music listening experiences and social participation. They shared their findings with the consortium partners as well as an external researcher (TS), who was not involved in the Musi-CI training to prevent bias and enhance the credibility of the findings. Additionally, they conducted a member check (Birt et al., 2016) among the Musi-CI training participants by sending all 37 participants a report of the analyses-outcomes; 24 of the participants replied and all respondents gave their agreement. The Musi-CI Handbook was written based on these findings, providing useful information about musical training for CI rehabilitation professionals.

## Results

The Musi-CI training was designed according to phase 1 of the NIHR-MRC framework, which, in phase 2, was modified as a result of the 3-cycle PAR process of obtaining input and feedback from CI users and CI rehabilitation professionals.

### Participants

All training sessions were very well attended, with an average attendance of 95% at the live sessions; 32 (86%) of all 37 participants completed the training. Reasons for quitting were very diverse: participant P11, an accomplished amateur pianist, left the training due to permanent disappointment with the CI's music sound whilst playing, P13 and P14 left the training due to severe problems with speech comprehension and P16 and P18 left the training due to the COVID-19 lockdown and subsequent online classes not satisfying their needs. A few participants attended multiple cycles. P12 – based on a strongly expressed wish to gain as much experience as possible with music – participated in all three cycles, and thus became a valuable ambassador. P6 and P9 only partially participated in cycle 1 due to personal circumstances and later re-joined in cycle 3.

### Start-End Questionnaires

The start-end questionnaires consisted of two questions. Table 3 shows the outcome of question 1: the categories of personal goals, goals stated at the start of the training, and goals attained at the end of each PAR cycle. The results show that personal goals were not easily achieved. Some participants noticed a discrepancy between their pre-training expectations and post-training achieved goals. They had only partially reached their goals or achieved different goals than expected beforehand. The most frequently mentioned personal goal was ‘music appreciation and recognition’. This included recognising familiar melodies. As Table 3 shows, 6 participants aimed for this goal, but only 3 partially attained that goal. More appreciation and enjoyment, on the contrary, were desired by 19 participants and fully attained by 18. An additional 2 participants partially reached this goal, with one of them not mentioning it as a goal in the Start-questionnaire.

Question 2 concerns participants’ level of motivation to participate in the Musi-CI training. At the start, most participants indicated that they were highly motivated and eager to bring music back into their lives. At the end of the Musi-CI training about half of the participants indicated that they had underestimated the effort it takes to start listening to music again and integrate the exercises into daily life. An instructor who could provide them with a variety of homework assignments was considered very helpful. The outcomes of the personal goals and motivation ratings analysis were used to improve the training programme in dialogue with participants.

*In-between evaluations.* After each PAR cycle, written feedback from participants and observations from CI rehabilitation professionals were collapsed into training categories relevant to modifying the training. A summary of these results is presented below.

### Feedback from CI Users, After Cycle 1

1. *Listening and watching*. All 12 participants were enthusiastic and pleasantly surprised by the impact of visual support, i.e., watching YouTube videos, during the training as well as at home. Even those participants who did not complete the training appreciated the addition of visuals to their music listening experience. “*Music videos are a revelation. When you watch them with focused attention, they work like subtitles to the music”.*
2. *Musical genres.* The CI users’ feedback indicates that a variety of exercises was highly valued. By separating music into its basic elements (beat, rhythm, pitch, timbre), ‘*chaotic, discordant musical sounds*’ (music they perceived as incomprehensible) were avoided. This made the presented music much more ‘*digestible’*. Also, a greater variety of musical genre was welcome because *‘we encounter all kinds of music in our daily lives’*.
3. *Rhythmic exercises*. The system of the Ronnie Gardiner Method (RGM) adapted clapping exercises was not as effective as we expected it to be. Participants expressed difficulties with doing these exercises at home.
4. *Singing*. The goal of singing together was to facilitate the process of pitch discrimination. Most participants were reluctant to sing even familiar nursery rhymes, convinced they would sing out of tune. By examining the singing process, participants concluded that singing is not suitable for improving pitch discrimination, due to their difficulties correctly perceiving pitch steps whilst singing. Nevertheless, this experience did provide some music enjoyment. “*I sang bim-bam, the others sang something else. We persevered and at the end it went quite well and it sounded good!”*
5. *Group discussions*. Participants indicated a need for peer support, sharing experiences of success with listening to music, and sharing experiences to refine their coping strategies.
6. *Homework*. In order to help participants to develop new listening habits, homework was sent via email after each lesson. Almost all participants stated that these homework assignments helped them to listen more often to music and to enjoy it more. “*I use the homework music as a nice start of exercising. After the prescribed homework I challenge myself with more and different types of music that I found, using my iPhone and streamer”.*

### Feedback from CI Rehabilitation Professionals, After Cycle 1

The CI rehabilitation professionals of the Consortium (CB, AG) and their direct colleagues, who conducted observations of the training groups, reported that the atmosphere in the lessons was open and communicative. They had the impression that peer contact was strikingly important for participants. They examined whether the difficulty level of the lessons optimally corresponded with the abilities of the different CI users.

### Modifications of the Musi-CI Training After Cycle 1 Leading to Musi-CI Design 2

After consulting with the consortium and taking the feedback on Cycle 1 into account, the training content was modified for Cycle 2 (see Table 4). To train pitch differences, we replaced the singing exercises with playing with 8 individual keys of a metallophone, constituting a major scale above middle C, hereinafter referred to as sound bars. Another reason to omit singing was the COVID-19 restrictions, which did not allow singing in a group setting at that time. To encourage music practice at home, we decided to develop tools for online exercises and deliberate at-home practice.

**Table 4.** Overview of the Adaptations to the Musi-CI Training, Design 2.

EXERCISES	Content of DESIGN 1	Content of DESIGN 2
Music listening exercises	Listening to instruments, mainly in classical music, using (YouTube) videos as visual support of music.	Listening to instruments across a wider range of musical genres, such as Pop and Jazz. Focus placed on ‘non-chaotic’, not overly complex music
	Listening to varying moods in music.	Listening to varying moods in music with the inclusion of music from TV and film.
Music making exercises	Exercises with beat and rhythm. Clapping exercises with symbols for movements, adapted from the Ronnie Gardener Method.	Exercises with beat and rhythm were improved with the use of the original RGM symbols, to emphasise correctly feeling the beat and moving in time. NMT clapping exercises to train divided and focused attention (like clapping circles) were added.
Singing	Singing exercises with well-known songs and rounds.	Singing exercises were replaced with sound bars (representing tone steps) to work on pitch differences and short melodies.
Group discussions	No specific plan for group discussions.	To focus more attention on the group discussions, a list of topics to discuss in the group was designed to support peer contact, social learning and experiences of self-efficacy.
Homework	A brief summary of the lessons	Minor adaptations to the structure of the homework e-mails were made.

### Feedback from CI Users After Cycle 2

1. *Musical genres*. All participants stated that variation in musical style is necessary, but insisted against the music being too complex: *“I am able to handle only one and maximally three instruments. Multi-instrument bands I experience as chaotic and too noisy; then I stop listening”.*
2. *Rhythmic exercises.* Participants’ experiences with the RGM exercises were positive. The choice of symbols was valued positively. However, the number of different symbols was perceived as too high. The rhythmic exercises derived from NMT facilitated more active music making and added wider variety to the lessons.
3. *Introduction of sound bars.* Practicing pitch and short melodies using the sound bars in playful exercises was a success. We observed that participants showed a level of frequency discrimination that exceeded both their and our expectations, which the participants also reported. This way of active music making was highly enjoyed. *“When I make music without attention it all sounds very chaotic because everybody is playing at the same time. When I start paying attention to my own ‘tick-tick sounds’, suddenly I hear the music as a whole. This sounds beautiful”.*
4. *Group discussions.* After discussing with the participants how to develop new listening habits, discussions about experiences of success and self-efficacy were integrated into the lesson content to stimulate social learning. This also stimulated exchange amongst participants about feelings of music loss.
5. *Online classes*. Due to COVID-19, in person classes were not possible in April and May 2020. During this period, the lessons were delivered as remote video classes and continued in smaller groups of 2 to 3 participants. The advantage of this was that we were able to maintain contact with the participants, contributing to the continuation of musical involvement. Despite many technological issues including image and sound synchronisation and/or sound quality of loudspeakers, the classes were appreciated, and most of the feedback from participants remained positive. *“Zoom is better than nothing, in person is 100% better”.*

### Feedback from CI Rehabilitation Professionals, After Cycle 2

According to their observations, the CI rehabilitation professionals of the consortium stated that the pacing of the lessons should be monitored and carefully adjusted to the capability of participants by e.g., altering the number of exercises when appropriate. Also, the exercises at the beginning of Cycle 2 were sometimes too difficult; this was registered and adjusted accordingly.

The speech and language therapists also observed that participants who were inexperienced CI users (less than 12 months CI experience), had more difficulty doing the assignments of the Musi-CI training. Therefore, in cycle 3 we changed the inclusion criterion from 6 months of minimal CI use to approximately 12 months, resulting in all 16 participants successfully completing the training.

### Modifications of the Musi-CI Training After Cycle 2, Leading to Musi-CI Design 3

**Figure 2.**
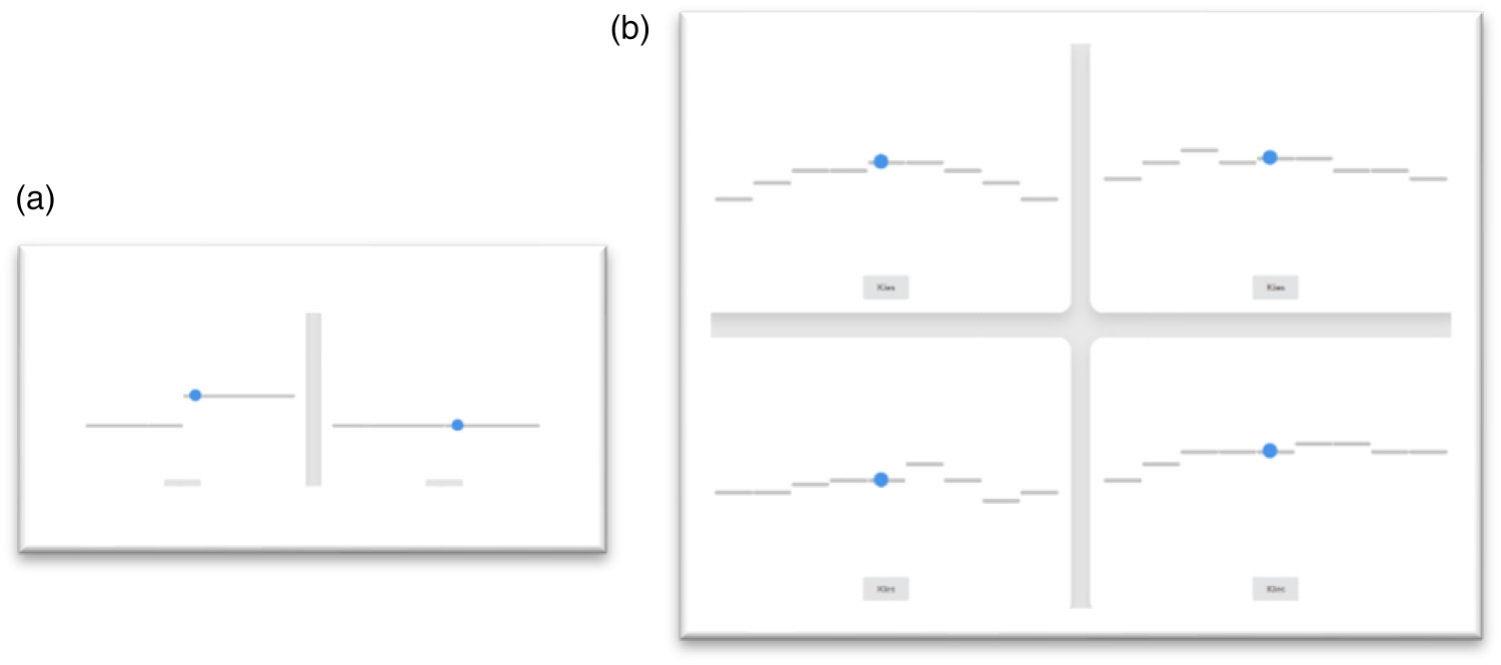
(a) Melody game, level 1. (b) Melody Game, level 7.

After consulting with the consortium, all exercises were improved in conformity with all input and feedback. We introduced online exercises of RGM that were suitable for homework. A major modification was the introduction of the Melody Game, a newly developed online training tool, to enhance deliberate practice at home. This game consists of 7 levels with exercises ranging from distinguishing two tones (level 1, Figure 2a) to distinguishing short melodies of 9 to 11 tones (level 7, Figure 2b). The auditory input of the game is represented by blue dots following short lines, visualising the melodic contours to supplement the learning process (Table 5). The discrimination-game is played by discerning which contours you hear.

**Table 5.** Overview of the Adaptations of the Musi-CI Training, Design 3.

EXERCISES	Content of DESIGN 2	Content of DESIGN 3
Music listening exercises	Listening to instruments across a wider range of musical genres, such as Pop and Jazz. Focus placed on ‘non-chaotic’, not overly complex music	Listening to music was enhanced with the use of music selected by the participants in lessons 5–8. They learned to browse music as a search strategy for personal preferences
	Listening to varying moods in music with the inclusion of music from TV and film	No changes
Music making exercises	Exercises with beat and rhythm were improved with the use of the original RGM symbols, to emphasise correctly feeling the beat and moving in time. NMT clapping exercises to train divided and focused attention (like clapping circles) were added.	RGM exercises were limited to 6 symbols. We introduced RGM online exercises, suitable for homework assignments. Exercises with dance rhythms like Bossa Nova were added. Rhythmics were extended with exercises for working memory (APT and MEMT, see Supplementary File 1)
Singing	Singing exercises were replaced with sound bars (representing tone steps) to work on pitch differences and short melodies.	The sound bars exercises were extended by utilising the Melody Game, an online tool for pitch training at home.
Group discussions	To focus more attention on the group discussions, a list of topics to discuss in the peer group was designed to support peer contact, social learning and experiences of self-efficacy.	To pay close attention to group discussions, the list of topics was adjusted, to include topics such as exploring music instead of recognising music.
Homework	Minor adaptations to the homework e-mails were made.	RGM-online exercises and the Melody Game were added

### Feedback from CI Users After Cycle 3

1. *Rhythmic exercises.* The RGM online exercises were well received, in part because they could easily be performed at home. We were driven by feedback to conduct these particular exercises during the lessons, with sufficient repetition. This increased participants’ confidence. “*When all the ‘clap-bang’ took too much time and I did not succeed so well, the lesson became dull and tiring. You need to keep reminding yourself of the higher aim”.*
2. *Melody Game.* Participants appreciated the inclusion of the Melody Game but found it difficult and sometimes even frustrating. “*You have to listen very carefully. I see the lines go up, but I hear something else. That makes it a really challenging game. But if I am successful, I feel really good about that.”*
3. *Group discussions.* During the group discussions we explored the topic of insufficient self-efficacy beliefs regarding listening to and enjoying music. Participants indicated that interaction with the trainer and each other turned out to be essential. Participants highly valued that their trainer is a CI user herself.

### Feedback from CI Rehabilitation Professionals, After Cycle 3

The CI rehabilitation professionals of the Musi-CI Consortium observed that participants were eager to exchange their experiences with music, their feelings of loss, and how to cope with them. To structure the discussion, we designed a list of topics to facilitate the exchange of experiences, social learning and self-efficacy beliefs. A CI professional stated: “*I saw CI users go from initially afraid of music, through curiosity about music to actively looking for new music. An impressive process.”*

### End-Evaluations: Focus Group Evaluations with CI Users After Each Cycle

Focus groups were conducted after each PAR cycle to examine the impact on music listening and social participation. Participants stated they missed music and that this caused them to feel grief. They reported that contact with fellow CI users was one of the more valuable features of the entire training. They considered it advantageous to come into contact so that they could exchange experiences about difficult, sometimes painful, situations; being unable to recognise formerly familiar melodies was particularly painful, and this pain was soothed by peer support. The Musi-CI training and the peer contact provided a start to listening to music again. It contributed to the CI users’ feelings of inclusion and increased engagement with music; participants stated they attended concerts again or were planning to do so, and some had arranged to restart their music lessons.

The new way of more attentive listening was valued as a strong contribution. Although we did not explicitly test general auditory perception, participants made spontaneous remarks about it. “*I am sure, really 100%, that I hear better in general”.* Working together and discussing the advantages and disadvantages of exercises stimulated the learning process, which is consistent with social learning theories (see Supplementary File 1).

“We share emotions together, because hearing loss is an underestimated, lonely handicap.”

### Summary of Perceived Effective Components of the Training After 3 PAR Cycles

We identified 7 themes from the thematic analysis representing the perceived effective components of the training. Table 6 shows the results including quotes from participants. Developing a new way of listening is an essential step for participants (1), with attention to listening and watching (2). Embracing paying attention to timbre (3), and to the mood of the music, like happy, sad or scary (4), is more effective than making sole efforts to recognise familiar songs or melodies. We realised that they were surprisingly capable of distinguishing different atmospheres and emotions and that they showed a high level of congruence on this.

**Table 6.** Perceived Effective Components by CI Participants of the Musi-CI Training.

1.	A new way of listening “*This is a new, different way of listening, more intense and with much more attention”. “It is exercising, making mistakes, and starting anew. Stimulate your concentration and get your brain started*”. “*The conscious, attentive way of listening is the best element of the training”.*
2.	Listening and watching “*Great, not all music in the YouTube videos is my style, but I get the chance to figure things out for myself; before I realise an hour has passed, watching and listening*”. “*Watching videos while listening is like subtitles with the music*”.
3.	Listening to instruments “*My way of listening has changed significantly. Music is slowly emerging from the ‘chaos of sound’. I even started recognising instruments*”. “*As a lover of hard rock music I’m fond of really loud music. But now I pay attention to the instruments. That makes listening to music with my CI more interesting and I hear all kinds of winds and guitars”.*
4.	Listening to emotion in music “*This listening activity invites me to feel what the music is doing to me. How it touches or irritates me. It supports me to discuss these emotions in our group*”. “*All music was about emotion to me. Folk music as well as classical music such as the Matthew Passion. But now it's emotional for me because it sounds so chaotic and distorted, I can hardly bring myself to listen. I still have a long way to go”.*
5.	Specific exercises with feeling beat and rhythm “*When I can feel the beat and can make movements accordingly, I understand music better*.” “*Since I’ve gotten used to paying attention to the feel of the beat, I’ve found myself softly clapping on my leg with all music I listen to. This makes music sound less ‘chaotic’ or ‘busy’.*
6.	Specific exercises with pitch and melody “*In the beginning I couldn’t tell apart any tones, but gradually I started to recognise what these differences sound like. Playing the sound bars together became more and more of a pleasure.”* “*I was working with the Melody Game. When I look at one of the pictures, symbolizing what you hear, then I hear that one, even if it's wrong. That's weird. I better listen with my eyes closed so as not to get disturbed by the visuals”.*
7.	Independent deliberate practice: the Melody Game “*I like working with the Melody Game, because you have to concentrate a lot, otherwise I tend to mix up higher and lower. It's frustrating when I think I’m right and it is wrong again…”* “*When working with the Melody Game I not only pay attention to high or low tones, but also whether they sound sharp and dull. This helps me to discriminate better”.*

Training to feel the beat proved helpful, because the body ‘responds’ to music through natural movement (5). Awareness of the beat is a means to engage with music. Actively working on pitch and short melodies together during classes by playing sound bars was challenging, but the challenge proved to be fun (6). The Melody Game formed a good addition to playing sound bars, providing an opportunity for independent, deliberate practice (7).

In conclusion, helping CI users to feel confident in their ability to listen to music attentively is an important step in treating their music aversion.

#### Musi-CI Training and Handbook 10

The final result of the iterative PAR process is the Musi-CI training itself. The Musi-CI Handbook 1.0 contains all exercises, the Musi-CI trainer's required qualifications, and all necessary practical conditions to conduct the Musi-CI training in a feasible manner (see programme guide at www.musi-ci.nl/english). This handbook makes the results of this study available for both future research and clinical practice.

## Discussion

In this study, the “Musi-CI training” and a Musi-CI Handbook 1.0 for CI rehabilitation professionals are developed according to a PAR approach. In this PAR approach there was intensive collaboration between the Musi-CI Consortium and the participating CI users. JV's experiential and professional knowledge as musician, CI user and initiator of the Musi-CI training, as well as the Musi-CI consortium's expertise were central to the co-design and conduct of the training. This broad interdisciplinary collaboration makes the Musi-CI project unique. JV's credibility with participants as trainer and role model is a contributing factor to the success of the Musi-CI training.

We learned from the focus group interviews that participants unanimously agreed that a Musi-CI trainer needs to have sufficient knowledge and experience working with music and the capacity to empathise with the musical disabilities and/or capabilities of CI users. Listening with a CI is so different from normal hearing that working with them requires consideration and compassion towards their feelings of loss and shame, e.g., being unable to recognise familiar music. This finding confirms the validity of our conceptual framework. However, this finding is difficult to generalise to new contexts. Sharing video recordings of JV and of other CI users experiencing success with this training may contribute to the motivation of future Musi-CI trainers and participants. To enhance transferability ongoing research is needed with different CI users, different trainers in a different contexts.

The study was based on previous studies reporting that musical listening training can result in improved music enjoyment (Fuller et al., 2018; Plant, 2015) and quality of life (Magele et al., 2022; Riley et al., 2018). The key elements of the Musi-CI training are discussed here (see Table 6).

### A New Way of Listening

Our findings show that CI users need to find a new way to listen to music. Former experiences with music (before CI use) are both an advantage (a musical foundation to fall back on) and a disadvantage (high expectations when listening to music). As shown in Tables 3 and 6, participants reported increased appreciation whilst listening attentively to music. They reported a positive impact on general audio perception, such as sound recognition, and on their social participation related to music, such as taking up music lessons or dancing classes and attending concerts, festivals, or parties. This could be considered a positive result of re-engaging with music (van Besouw et al., 2015).

### Recognition of Familiar Melodies

Recognition of familiar melodies is often beyond the scope of CI users’ abilities as melody is conveyed poorly by the CI. It is important to set realistic expectations for the new ways of listening and to discuss this during classes. As Prevoteau et al. (2018) stated, recognition could be poor despite good discernment qualities, but enjoyment is still possible even without recognition. This is a crucial, but also often painful subject of discussion during training. This underscores the need for a safe, comfortable relationship between trainer and participants.

### Musical Genres

Preferences of musical genres differ from one individual to the other. A variety of musical genres gives participants the opportunity to interact with music they are not familiar with but may appreciate nonetheless. The literature often states that familiar music is most effective (Magele et al., 2022; Plant, 2015). This might be true, but on occasion our participants showed high expectations regarding familiar music and felt very disappointed when unable to recognise it. Unfamiliar music is more likely to be approached with an open mind. Hang Drum music and less complex classical music are good examples.

### Beat and Rhythm

As shown by Hsiao and Gfeller (2012) it is preferable to work with the basic elements of music, i.e., beat and rhythm, pitch and melody, and timbre as the specific sounds of instruments. While working with the beat of music we found that participants need to learn to ‘feel’ this. Although, e.g. Phillips-Silver et al. (2015) showed that CI users perceive beat and rhythm (nearly) as well as normal-hearing people, we learned during our training sessions that it is beneficial to train CI users to feel the beat by physically synchronising with it, and to start applying this newly developed skill to different kinds of music. Beat is the underlying pulse of a piece of music – its beating heart – and an easily perceived structural baseline. As soon as CI users were sufficiently able to do so, they mentioned music sounding less “chaotic”, overwhelming and blurry. This awareness contributed to reduced music avoidance*.*

### Pitch and Melody

Participants valued training pitch and melody very highly. This could be a result of the ever-present wish to get better at following melody or the hope of restoring recognition of formerly familiar melodies. Pitch and melody were the most difficult aspects of music for the Musi-CI participants to be identified, and our results are supported by reports e.g., Jiam et al. (2019), Magele et al. (2022) and van Heteren et al. (2022), the latter reporting that only a minority of CI users (7/19) could robustly discriminate between tones differing by a semitone.

### Independent Deliberate Practice

The Melody Game (conceived during cycle 1, developed during cycle 2, and tested during cycle 3) is an online tool for independently, deliberately practicing pitch perception and short melody tracking at home. This exercise could aid in improving pitch discrimination (Smith et al., 2017), which could in turn contribute to an improved ability to follow a melodic line. Participants who cooperated with the design team during the try-out in Cycle 3 reported that the Melody Game was effective at training discrimination between pitches or melody fragments. However, the Melody Game's exercises lacked sufficient feedback, which reduced the experience of gratification, and did not encourage people to practise longer. Therefore, further development is required.

### Listening to Instruments

Listening with focused attention for the subtlest timbre differences between instruments that the CI is able to process resulted in improved music enjoyment. Participants stated that the sound of many solo instruments from all musical instrument families (strings, guitars, piano, woodwinds, brass, drums) became more enjoyable, with the notable exception of the organ. This is in consonance with the literature that indicates that instrument recognition is trainable and most effective with spaced rehearsals (Gfeller et al., 2019b), added visual cues (Chari et al., 2020; Plant, 2015), in an active training with focus and attention (Smith et al., 2017) and reduced complexity to enhance the listening experiences (Prevoteau et al., 2018). As the complexity of music is increased, with more instruments playing simultaneously, music appreciation decreases. Developing a stepwise approach to coach the average CI user from enjoying solo music to appreciating more complex music, could be a valuable subsequent contribution to the literature and deserves further attention.

### Listening to Emotions

To assist participants in taking a step towards appreciating more complex music we attempted to make them aware of the emotions conveyed by music. These emotions, which contribute to the mood of the music, flow together smoothly in most pieces of music. Being able to absorb this flow of emotions may contribute to music enjoyment. The participants were exposed to musical fragments that expressed different emotions such as happiness, fear, sadness, and peace, according to Ambert-Dahan et al. (2015). The emotions the participants did perceive were mostly consistent with each other. These results are in line with the results from Rosslau et al. (2012). That study described discerning emotions from a piece of music as experiencing musical arousal and concluded that this is a well-preserved ability in CI users.

### In-Person Training

Despite the degraded perception of music with a CI compared to normal hearing, some CI users can acclimate to the ‘new sound’ of the CI and continue listening to music while other CI users avoid listening to music. Enjoyment and subjective appreciation do not always reflect perceptual abilities, because enjoyment is also dependent on personal, situational, and emotional variables, cf. Riley et al. (2018). Our study shows that participants highly valued the peer contact, interaction, and discussions that were made possible during the in-person, group training sessions. We considered the potential of online practice modules as a valuable addition, but not a replacement of the in-person training sessions in contrast to van Besouw et al. (2015) who proposed training exclusively online.

### Inclusion Criteria

The outcome that the Musi-CI training was too difficult and/or too overwhelming for participants in the early phase of CI rehabilitation necessitates the formulation of clear inclusion criteria based on a strong rationale before testing the training on a larger group of CI-users. Inclusion criteria should include a stable audiological setting and a stable level of speech perception that allows for optimal group participation.

### Population Bias

The positive outcomes of the training on music listening, in general, are remarkable, but likely biased, because they are based only on the experiences of this select, non-representative test group. As most participants were self-registered, they may have been more motivated to attend Musi-CI training than the average CI user. This warrants a follow-up study, deploying qualitative and quantitative research into the effective factors of the Musi-CI training (phases 3 and 4 of the MRC framework). Outcomes of follow-up studies may also contribute to answering the question of how this musical listening training can be integrated into the current standard of care in CI rehabilitation.

### Trainer Bias

Because JV was a role model for the participating CI users, it remains unclear what her influence was on the outcomes. Although we have identified the critical success characteristics of the training to enhance its accessibility for other trainers, further research is required to determine if this training could be conducted by individuals/trainers who are not both musician and CI user.

## Conclusion

Musi-CI training is challenging but feasible for CI users who have approximately 12 months experience listening with their CI. In a participatory and iterative approach with CI users and CI rehabilitation professionals, it proved to be possible to develop a training that satisfies all underlying theoretical principles; namely facilitating experiences of success and self-efficacy, and contextual, collaborative group learning. The eclectic approach of the training, with a variety of music listening and music making exercises, was deemed as positive and valuable by CI users and CI rehabilitation professionals alike. The handbook makes the results of our study available for both research and clinical practice. This requires consequent quantitative studies, to examine the effective factors of the Musi-CI training (phases 3 and 4 of the NIHR-MRC framework).

## Supplemental Material

sj-docx-1-tia-10.1177_23312165231198368 - Supplemental material for Development of the Musi-CI Training, A Musical Listening Training for Cochlear Implant Users: A Participatory Action Research Approach Click here for additional data file. Supplemental material, sj-docx-1-tia-10.1177_23312165231198368 for Development of the Musi-CI Training, A Musical Listening Training for Cochlear Implant Users: A Participatory Action Research Approach by Joke Veltman, Marjo J.M. Maas, Cilia Beijk, Adinda Y.M. Groenhuis, Huib Versnel, Constance Vissers, Wendy J. Huinck and Alexander E. Hoetink in Trends in Hearing

sj-docx-2-tia-10.1177_23312165231198368 - Supplemental material for Development of the Musi-CI Training, A Musical Listening Training for Cochlear Implant Users: A Participatory Action Research Approach Click here for additional data file. Supplemental material, sj-docx-2-tia-10.1177_23312165231198368 for Development of the Musi-CI Training, A Musical Listening Training for Cochlear Implant Users: A Participatory Action Research Approach by Joke Veltman, Marjo J.M. Maas, Cilia Beijk, Adinda Y.M. Groenhuis, Huib Versnel, Constance Vissers, Wendy J. Huinck and Alexander E. Hoetink in Trends in Hearing

## References

[bibr1-23312165231198368] Abma T. Banks S. Cook T. Dias S. Madsen W. Springett J. Wright M. T. (2019). Participatory research for health and social well-being. Springer International Publishing. 10.1007/978-3-319-93191-3

[bibr2-23312165231198368] Abma T. A. Broerse J. E. W. (2010). Patient participation as dialogue: Setting research agendas. Health Expectations, 13(2), 160–173. 10.1111/j.1369-7625.2009.00549.x 20536537 PMC5060528

[bibr3-23312165231198368] Ambert-Dahan E. Giraud A.-L. Sterkers O. Samson S. (2015). Judgment of musical emotions after cochlear implantation in adults with progressive deafness. Frontiers in Psychology, 6, 10.3389/fpsyg.2015.00181 PMC4357245 25814961

[bibr4-23312165231198368] Baddeley A. (2010). Working memory. Current Biology, 20(4), R136–R140. 10.1016/j.cub.2009.12.014 20178752

[bibr5-23312165231198368] Bandura A. Locke E. A. (2003). Negative self-efficacy and goal effects revisited. Journal of Applied Psychology, 88(1), 87–99. 10.1037/0021-9010.88.1.87 12675397

[bibr6-23312165231198368] Bergold J. Thomas S. (2012). Participatory research methods: A methodological approach in motion. Historical Social Research, 37(4), 191–222. ISSN: 01726404

[bibr7-23312165231198368] Birt L. Scott S. Cavers D. Campbell C. Walter F. (2016). Member checking: A tool to enhance trustworthiness or merely a nod to validation? Qualitative Health Research, 26(13), 1802–1811. 10.1177/1049732316654870 27340178

[bibr8-23312165231198368] Chari D. A. Barrett K. C. Patel A. D. Colgrove T. R. Jiradejvong P. Jacobs L. Y. Limb C. J. (2020). Impact of auditory-motor musical training on melodic pattern recognition in cochlear implant users. Otology & Neurotology, 41(4), e422–e431. 10.1097/MAO.0000000000002525 32176126

[bibr9-23312165231198368] Cross I. (2009). La naturaleza evolucionista de la significacién musical. Musicae Scientiae, 13(2 SUPPL.), 179–200. 10.1177/1029864909013002091

[bibr10-23312165231198368] Deci E. L. Ryan R. M. (2000). The “what” and “why” of goal pursuits: Human needs and the self-determination of behavior. Psychological Inquiry, 11(4), 227–268. 10.1207/S15327965PLI1104_01

[bibr11-23312165231198368] Dorman M. F. Natale S. C. Baxter L. Zeitler D. M. Carlson M. L. Lorens A. Skarzynski H. Peters J. P. M. Torres J. H. Noble J. H. (2020). Approximations to the voice of a cochlear implant: Explorations with single-sided deaf listeners. Trends in Hearing, 24, 233121652092007. 10.1177/2331216520920079 PMC7225791 32339072

[bibr12-23312165231198368] Driscoll V. (2012). The effects of training on recognition of musical instruments by adults with cochlear implants. Seminars in Hearing, 33(04), 410–418. 10.1055/s-0032-1329230 23503992 PMC3595548

[bibr13-23312165231198368] Driscoll V. D. Oleson J. Jiang D. Gfeller K. (2009). Effects of training on recognition of musical instruments presented through cochlear implant simulations. Journal of the American Academy of Audiology, 20(1), 71–82. 10.3766/jaaa.20.1.7 19927684 PMC2784659

[bibr14-23312165231198368] Dritsakis G. van Besouw R. M. O’ Meara A. (2017). Impact of music on the quality of life of cochlear implant users: A focus group study. Cochlear Implants International, 18(4), 207–215. 10.1080/14670100.2017.1303892 28326996

[bibr15-23312165231198368] Fuller C. D. Galvin J. J. Maat B. Başkent D. Free R. H. (2018). Comparison of two music training approaches on music and speech perception in cochlear implant users. Trends in Hearing, 22, 1–22. 10.1177/2331216518765379 PMC5894911 29621947

[bibr16-23312165231198368] Galvin J. J. Fu Q.-J. Nogaki G. (2007). Melodic contour identification by cochlear implant listeners. Ear & Hearing, 28(3), 302–319. 10.1097/01.aud.0000261689.35445.20 17485980 PMC3627492

[bibr17-23312165231198368] Gfeller K. Driscoll V. Schwalje A. (2019a). Adult cochlear implant Recipients’ perspectives on experiences with music in everyday life: A multifaceted and dynamic phenomenon. Frontiers in Neuroscience, 13(November), 1–19. 10.3389/fnins.2019.01229 31824240 PMC6882382

[bibr18-23312165231198368] Gfeller K. Guthe E. Driscoll V. Brown C. J. (2015). A preliminary report of music-based training for adult cochlear implant users: Rationales and development. Cochlear Implants International, 16(S3), S22–S31. 10.1179/1467010015Z.000000000269 26561884 PMC4646703

[bibr19-23312165231198368] Gfeller K. Mallalieu R. M. M. Mansouri A. McCormick G. O’Connell R. B. Spinowitz J. Gellinek Turner B. (2019b). Practices and Attitudes That Enhance Music Engagement of Adult Cochlear Implant Users. Frontiers in Neuroscience, 13(December). 10.3389/fnins.2019.01368 PMC6937904 31920520

[bibr20-23312165231198368] Gfeller K. Olszewski C. Rychener M. Sena K. Knutson J. Witt S. Macpherson B. (2005). Recognition of “real-world” musical excerpts by CI recipients and normal hearing adults. Ear & Hearing, 26(3), 237–250. 10.1097/00003446-200506000-00001 15937406

[bibr21-23312165231198368] Hsiao F. Gfeller K. (2012). Music perception of cochlear implant recipients with implications for music instruction. Update: Applications of Research in Music Education, 30(2), 5–10. 10.1177/8755123312437050 23469365 PMC3587135

[bibr22-23312165231198368] Hutter E. Argstatter H. Grapp M. Plinkert P. K. (2015). Music therapy as specific and complementary training for adults after cochlear implantation: A pilot study. Cochlear Implants International, 16(S3), S13–S21. 10.1179/1467010015Z.000000000261 26047068

[bibr23-23312165231198368] Hutter E. Grapp M. Argstatter H. (2016). Musiktherapie bei erwachsenen CI-trägern. HNO, 64(12), 880–890. 10.1007/s00106-016-0279-7 27837214

[bibr24-23312165231198368] Jiam N. Deroche M. Jiradejvong P. Limb C. (2019). A Randomized Controlled Crossover Study of the Impact of Online Music Training on Pitch and Timbre perception in Cochlear Implant Users. Journal of the Association for Research in Otolaryngology, 20(3), 247–262. 10.1007/s10162-018-00704-0 30815761 PMC6514036

[bibr25-23312165231198368] Jiam N. T. Caldwell M. T. Limb C. J. (2017). What does music sound like for a cochlear implant user? Otology and Neurotology, 38(8), e240–e247. 10.1097/MAO.0000000000001448 28806333

[bibr26-23312165231198368] Jiam N. T. Deroche M. L. Jiradejvong P. Limb C. J. (2019). A randomized controlled crossover study of the impact of online music training on pitch and timbre perception in cochlear implant users. Journal of the Association for Research in Otolaryngology, 20(3), 247–262. 10.1007/s10162-018-00704-0 30815761 PMC6514036

[bibr27-23312165231198368] Li L. C. Grimshaw J. M. Nielsen C. Judd M. Coyte P. C. Graham I. D. (2009). Use of communities of practice in business and health care sectors: A systematic review. Implementation Science, 4(1). 10.1186/1748-5908-4-27 PMC2694761 19445723

[bibr28-23312165231198368] Limb C. J. (2006). Cochlear implant-mediated perception of music. Current Opinion in Otolaryngology and Head and Neck Surgery, 14(5), 337–340. 10.1097/01.moo.0000244192.59184.bd 16974148

[bibr29-23312165231198368] Looi V. She J. (2010). Music perception of cochlear implant users: A questionnaire, and its implications for a music training program. International Journal of Audiology, 49(2), 116–128. 10.3109/14992020903405987 20151886

[bibr30-23312165231198368] Maas M. J. M. van Dulmen S. A. Sagasser M. H. Heerkens Y. F. van der Vleuten C. P. M. Nijhuis-van der Sanden M. W. G. van der Wees P. J. (2015). Critical features of peer assessment of clinical performance to enhance adherence to a low back pain guideline for physical therapists: A mixed methods design. BMC Medical Education, 15(1), 203. 10.1186/s12909-015-0484-1 26563246 PMC4643538

[bibr31-23312165231198368] Magele A. Wirthner B. Schoerg P. Ploder M. Sprinzl G. M. (2022). Improved Music Perception after Music Therapy Following Cochlear Implantation in the Elderly Population. Journal of Personalized Medicine, 12(3), 10.3390/jpm12030443 PMC8951547 35330442

[bibr32-23312165231198368] Norman G. Bordage G. Page G. Keane D. (2006). How specific is case specificity? Medical Education, 40(7), 618–623. 10.1111/j.1365-2929.2006.02511.x 16836533

[bibr33-23312165231198368] Patel A. D. (2011). Why would musical training benefit the neural encoding of speech? The OPERA hypothesis. Frontiers in Psychology, 2, 10.3389/fpsyg.2011.00142 PMC3128244 21747773

[bibr34-23312165231198368] Patel A. D. (2014). Can nonlinguistic musical training change the way the brain processes speech? The expanded OPERA hypothesis. In Hearing research (Vol. 308, pp. 98–108). Elsevier. 10.1016/j.heares.2013.08.011 24055761

[bibr35-23312165231198368] Peters J. P. M. Wendrich A. W. van Eijl R. H. M. Rhebergen K. S. Versnel H. Grolman W. (2018). The sound of a cochlear implant investigated in patients with single-sided deafness and a cochlear implant. Otology & Neurotology, 39(6), 707–714. 10.1097/MAO.0000000000001821 29889780

[bibr36-23312165231198368] Petersen B. Mortensen M. v. Gjedde A. Vuust P. (2009). Reestablishing speech understanding through musical ear training after cochlear implantation. Annals of the New York Academy of Sciences, 1169(1), 437–440. 10.1111/j.1749-6632.2009.04796.x 19673820

[bibr37-23312165231198368] Phillips-Silver J. Toiviainen P. Gosselin N. Turgeon C. Lepore F. Peretz I. (2015). Cochlear implant users move in time to the beat of drum music. Hearing Research, 321, 25–34. 10.1016/j.heares.2014.12.007 25575604

[bibr38-23312165231198368] Plant G. (2015). Musical FAVORS: Reintroducing music to adult cochlear implant users. Cochlear Implants International, 16(Suppl 3), S5–S12. 10.1179/1467010015Z.000000000271 26561887

[bibr39-23312165231198368] Pohl P. (2018). The Ronnie Gardiner method: An innovative music-based intervention for neurological rehabilitation - theoretical background and contemporary research with focus on Parkinson’s desease. Neurophysiology and Rehabilitation, 1(1), 32–37. https://doi.org/10.33805/2641-8991.111

[bibr40-23312165231198368] Prevoteau C. Chen S. Y. Lalwani A. K. (2018). Music enjoyment with cochlear implantation. Auris, Nasus, Larynx, 45(5), 895–902. 10.1016/j.anl.2017.11.008 29519690

[bibr41-23312165231198368] Riley P. E. Ruhl D. S. Camacho M. Tolisano A. M. (2018). Music appreciation after cochlear implantation in adult patients: A systematic review. Otolaryngology–Head and Neck Surgery, 158(6), 1002–1010. 10.1177/0194599818760559 29484920

[bibr42-23312165231198368] Rosslau K. Spreckelmeyer K. N. Saalfeld H. Westhofen M. (2012). Emotional and analytic music perception in cochlear implant users after optimizing the speech processor. Acta Oto-Laryngologica, 132(1), 64–71. 10.3109/00016489.2011.619569 22026456

[bibr43-23312165231198368] Skivington K. Matthews L. Simpson S. A. Craig P. Baird J. Blazeby J. M. Boyd K. A. Craig N. French D. P. McIntosh E. Petticrew M. Rycroft-Malone J. White M. Moore L. (2021). A new framework for developing and evaluating complex interventions: Update of medical research council guidance. BMJ, n2061. 10.1136/bmj.n2061 34593508 PMC8482308

[bibr44-23312165231198368] Smith L. Bartel L. Joglekar S. Chen J. (2017). Musical rehabilitation in adult cochlear implant recipients with a self-administered software. Otology and Neurotology, 38(8), e262–e267. 10.1097/MAO.0000000000001447 28806336

[bibr45-23312165231198368] Swaminathan J. Mason C. R. Streeter T. M. Best V. Kidd G. Jr Patel A. D. (2015). Musical training, individual differences and the cocktail party problem. Scientific Reports, 5(1), 11628. 10.1038/srep11628 26112910 PMC4481518

[bibr46-23312165231198368] Thaut M. H. Gardiner J. C. Holmberg D. Horwitz J. Kent L. Andrews G. Donelan B. McIntosh G. R. (2009). Neurologic music therapy improves executive function and emotional adjustment in traumatic brain injury rehabilitation. Annals of the New York Academy of Sciences, 1169, 406–416. 10.1111/j.1749-6632.2009.04585.x 19673815

[bibr47-23312165231198368] Thaut M. H. Hoemberg V. (2014). Handbook of neurologic music therapy. In Handbook of neurologic music therapy. Oxford University Press. http://sfx.nelliportaali.fi/sfxlcl3?url_ver=Z39.88-2004&rft_val_fmt=info:ofi/fmt:kev:mtx:book&genre=book&sid=ProQ:PsycINFO&atitle=&title=Handbook+of+neurologic+music+therapy.&issn=&date=2014-01-01&volume=&issue=&spage=372&au=&isbn=9780199695461&jtitle=&bt

[bibr48-23312165231198368] Vaismoradi M. Jones J. Turunen H. Snelgrove S. (2016). Theme development in qualitative content analysis and thematic analysis. Journal of Nursing Education and Practice, 6(5). 10.5430/jnep.v6n5p100

[bibr49-23312165231198368] van Besouw R. M. Nicholls D. R. Oliver B. R. Hodkinson S. M. Grasmeder M. L. (2014). Aural rehabilitation through music workshops for cochlear implant users. Journal of the American Academy of Audiology, 25(04), 311–323. 10.3766/jaaa.25.4.3 25126679

[bibr50-23312165231198368] van Besouw R. M. Oliver B. R. Grasmeder M. L. Hodkinson S. M. Solheim H. (2016). Evaluation of an interactive music awareness program for cochlear implant recipients. Music Perception, 33(4), 493–508. https://doi.org/https://www.jstor.org/stable/26417433

[bibr51-23312165231198368] van Besouw R. M. Oliver B. R. Hodkinson S. M. Polfreman R. Grasmeder M. L. (2015). Participatory design of a music aural rehabilitation programme. Cochlear Implants International, 16(S3), S39–S50. 10.1179/1467010015Z.000000000264 26561886

[bibr52-23312165231198368] Vandali A. Sly D. Cowan R. van Hoesel R. (2015). Training of cochlear implant users to improve pitch perception in the presence of competing place cues. Ear and Hearing, 36(2), e1–e13. 10.1097/AUD.0000000000000109 25329372

[bibr53-23312165231198368] van Heteren J. A. A. Vonck B. M. D. Stokroos R. J. Versnel H. Lammers M. J. W. (2022). The acoustic change complex compared to hearing performance in unilaterally and bilaterally deaf cochlear implant users. Ear and Hearing, 43, 1783–1799. 10.1097/AUD.0000000000001248 35696186 PMC9592183

[bibr54-23312165231198368] Vickers D. Salorio-Corbetto M. Driver S. Rocca C. Levtov Y. Sum K. Parmar B. Dritsakis G. Flores J. Jiang D. Mahon M. Early F. Van Zalk N. Picinali L. (2021). Involving Children and Teenagers with Bilateral Cochlear Implants in the Design of the BEARS (Both EARS) Virtual Reality Training Suite Improves Personalisation. Frontiers in Digital Health. 10.3389/fdgth.2021.759723 PMC8637804 34870270

[bibr55-23312165231198368] Visser C. (2010). Self-Determination theory meets solution-focused change: Autonomy, competence and relatedness support in action. InterAction - The Journal of Solution Focus in Organisations, 2(1), 7–26.

