# Supplementary material for: Development of the Musi-CI Training, A Musical Listening Training for Cochlear Implant Users: A Participatory Action Research Approach
Source: Trends Hear. 2023 Sep 12;27:23312165231198368. doi: 10.1177/23312165231198368 (PMC10496489; doi:10.1177/23312165231198368)
Supplement: sj-docx-1-tia-10.1177_23312165231198368 - Supplemental material for Development of the Musi-CI Training, A Musical Listening Training for Cochlear Implant Users: A Participatory Action Research Approach [file sj-docx-1-tia-10.1177_23312165231198368.docx]

Appendix Supplementary file 1, Professional Framework

| Theory | Characteristics | Operationalization of characteristics |
| --- | --- | --- |
| Music Education Theory (ABRSM, The Associated Board of the Royal Schools of Music, 2011) | ABRSM offers a comprehensive system of exercises and tests of increasing difficulty for amateur musicians. | The varied exercises add active elements to music listening by practicing the perception of (small) differences in rhythm, timbre or/and melody. This supports listening to music with focused attention. |
|  |  |  |
| Auditory Working Memory (Baddeley, 2010)  Mental Flexibility (Thaut et al., 2009)  Neurologic Music Therapy (NMT)  (Thaut & Hoemberg, 2014) | Training of auditory attention and working memory, using Auditory Perception Training (**APT**), Musical Attention Control Training (**MACT**), Musical Executive Function Training (**MEFT**), Musical Mnemonics Training (**MMT**), and Musical Echoic Memory Training (**MEMT**) | All exercises support active music making. **APT** focuses on perception of rhythm (clapping exercises) and pitch differences (soundbar exercises). (CI users are part of the target population).  **MACT** works with all kinds of clapping exercises to strengthen focused, divided and sustained attention.  **MEFT** works with unpredictable turn-taking to support mental flexibility.  **MMT** provides a method to better remember rhythms by adding text. Training of attention and working memory might be effective in supporting understanding of speech.  **MEMT** provides exercises to better hold auditory information and to strengthen working memory (CI users are part of the target population). |
|  |  |  |
| Ronnie Gardiner Method (RGM)  (Pohl, 2018) | Multi-sensory training, stimulating mobility, reading, speech, body image, self-esteem, balance, memory, motor skills, concentration, and social skills. | RGM is an easily accessible training method using complex clapping exercises that are meant to be synchronized to the beat of music. These multi-sensory exercises activate certain cortical regions that are involved in the execution of the exercises, including the auditory cortex. These exercises work well as homework assignments, via [www.rgm-online.nl](http://www.rgm-online.nl/). |
