# Supplementary material for: Development of the Musi-CI Training, A Musical Listening Training for Cochlear Implant Users: A Participatory Action Research Approach
Source: Trends Hear. 2023 Sep 12;27:23312165231198368. doi: 10.1177/23312165231198368 (PMC10496489; doi:10.1177/23312165231198368)
Supplement: sj-docx-2-tia-10.1177_23312165231198368 - Supplemental material for Development of the Musi-CI Training, A Musical Listening Training for Cochlear Implant Users: A Participatory Action Research Approach [file sj-docx-2-tia-10.1177_23312165231198368.docx]

Supplementary File 2, Conceptual Framework

| Theory | Characteristics | Operationalisation of characteristics |
| --- | --- | --- |
| Self-Determination theory  (Deci & Ryan, 2000)  Progress focused approach  (Visser, 2010) | Supporting feelings of autonomy, competence, and connectedness to enhance self-efficacy beliefs.  Supporting self-direction and self-management. | Conscious goal setting, focus on positive behavioural change.  Focus on abilities instead of impairments, strengthening positive experiences. |
|  |  |  |
| Social constructivist learning theory  (Norman et al., 2006) | Contextual learning, collaborative learning, active participation. | Presenting a variety of everyday music in different styles, adapted to the experiences and preferences of the CI users.  Stimulating exchange between participants about experiences with music and their way of coping.  Stimulating active participation in music making and music listening. |
|  |  |  |
| Social cognitive learning theory  (Bandura & Locke, 2003) | Enhancing the development of self-efficacy beliefs, by performing the new behaviour and experiencing the consequences of that behaviour (mastery experience).  Observing the behaviour of others and the consequences of that behaviour (vicarious experience). | Presenting a variety of everyday music in different styles, adapted to the experiences and preferences of the CI users.  Stimulating exchange between participants about experiences with music and their way of coping.  Stimulating active participation in music making and music listening. |
|  |  |  |
| Situated learning theory  (Li et al., 2009) | Learning in the context of daily life to bridge the gap between learning context and application context | Delivering the programme within the community of CI users, who share the same problems and interests. |

Conceptual model for the Musi-CI training, based on learning and behavior change theories, adapted from Maas et al. (2015).
